# Supplementary material for: On-chip coherent conversion of photonic quantum entanglement between different degrees of freedom
Source: Nat Commun. 2016 Jun 20;7:11985. doi: 10.1038/ncomms11985 (PMC4915126; doi:10.1038/ncomms11985)
Supplement: Supplementary Information — Supplementary Figures 1-7 [file ncomms11985-s1.pdf]

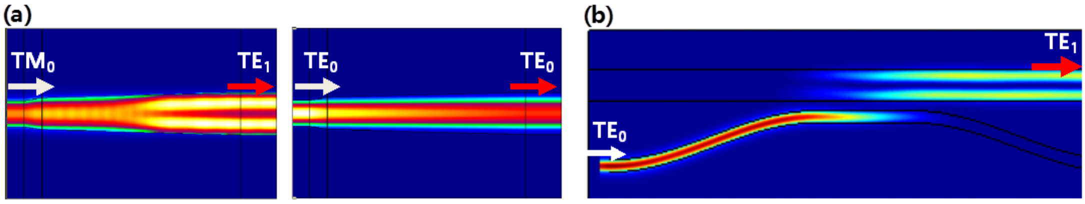


**Supplementary Figure 1**: **Simulated light propagation in the mode converter and mode multiplexer.** (a) The simulated light propagation in the mode converter based on an adiabatic taper, which can convert photons in TE and TM polarizations into the $TE_0$ and $TE_1$ modes, respectively. (b) The simulated light propagation in the mode multiplexer based on an asymmetric directional coupler, which can convert photons in upper path and lower path into the $TE_0$ and $TE_1$ modes, respectively. The simulation tools include the Fimmprop (PhotoDesign, Oxford, UK) employing an eigenmode expansion and the matching method and Lumerical software (Lumerical Solutions, Inc. London, UK) with the three-dimensional time-domain finite-difference (3DFDTD) method.

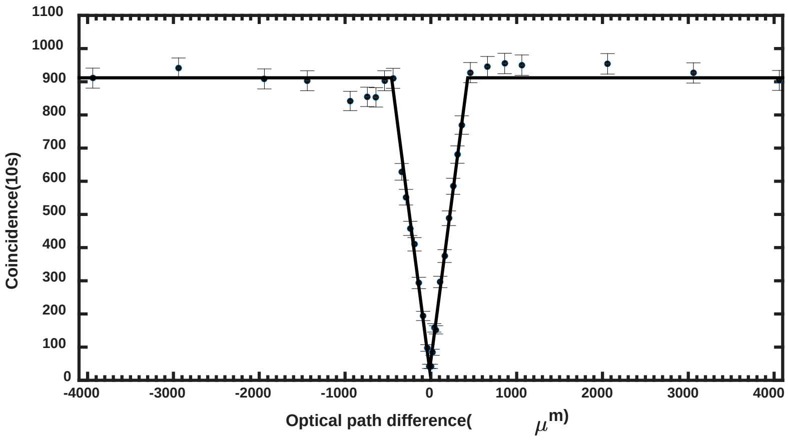


**Supplementary Figure 2**: **Hong-Ou-Mandel interference of photon pair source.** It was measured using a fibre 50:50 beam-splitter (BS). The central wavelength here is 1558nm; The Hong-Ou-Mandel (HOM) interference visibility is 96.3 ± 2.8% (96.5 ± 2.8% with background subtraction) and the optical coherence length is

$l_c$=448.7±19.8μm. So we can get the photons bandwidth is $\Delta\lambda = \frac{1.39}{\pi l_c}\lambda^2 = 2.4\text{nm}$.

The experimental data are fitted with a triangle function. Error bar comes from the Poisson statistical distribution.

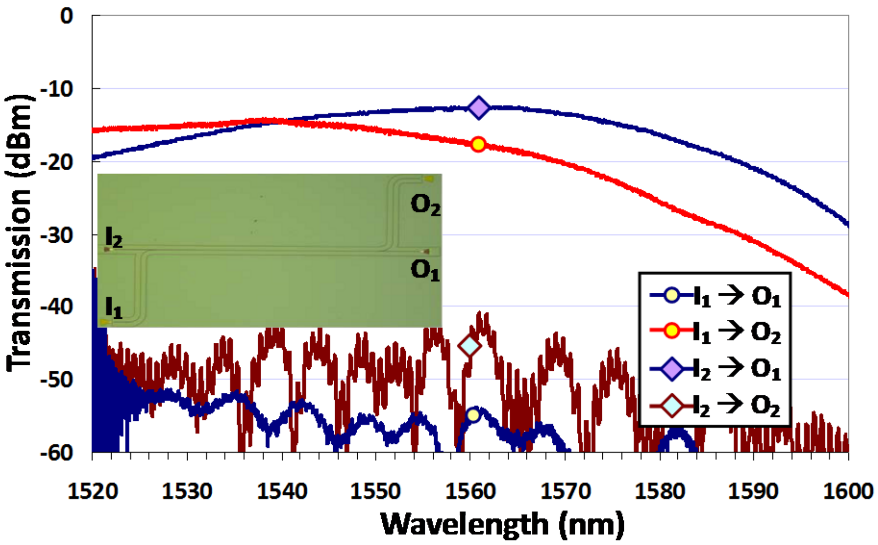


**Supplementary Figure 3**: **The microscope picture and the result of laser light test for sample 1.** The coupling loss was 12.5dB and crosstalk was -30dB at 1560nm when light was coupled in at TE-type grating, while coupling loss was 17dB and crosstalk was -30dB at 1560nm when light was coupled in at TM-type grating.

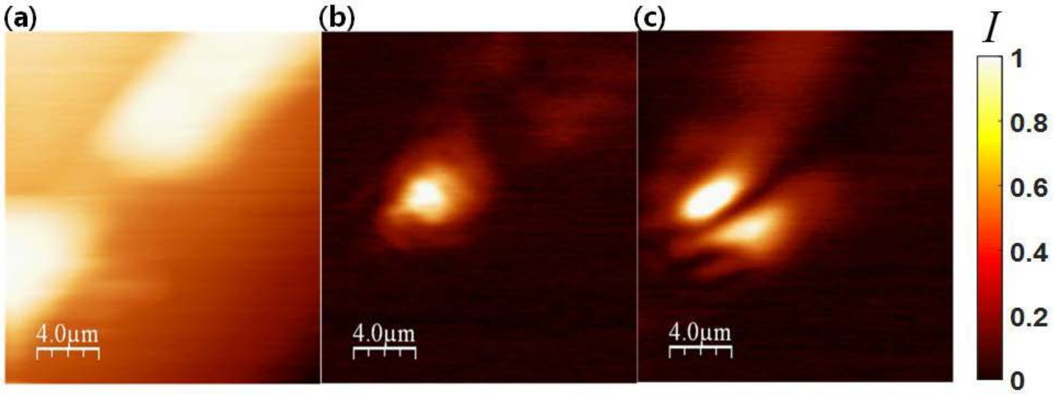


**Supplementary Figure 4**: **Near field scanning microscope (NSOM) measurements for sample 1.** (a) The height image of the multimode waveguide. Because of the protective layer on the waveguide, the waveguide width that we measured was about 4$\mu$m, much wider than the Silicon waveguide (750nm). (b) The energy distribution $I$ at the end of the multimode waveguide when we input laser beam at TE input port. (c) The energy distribution at the end of the multimode waveguide when input at TM input port. These results prove unambiguously that our polarization-dependent mode converter can convert laser beam in TE and TM polarizations into the $TE_0$ and $TE_1$ modes, respectively

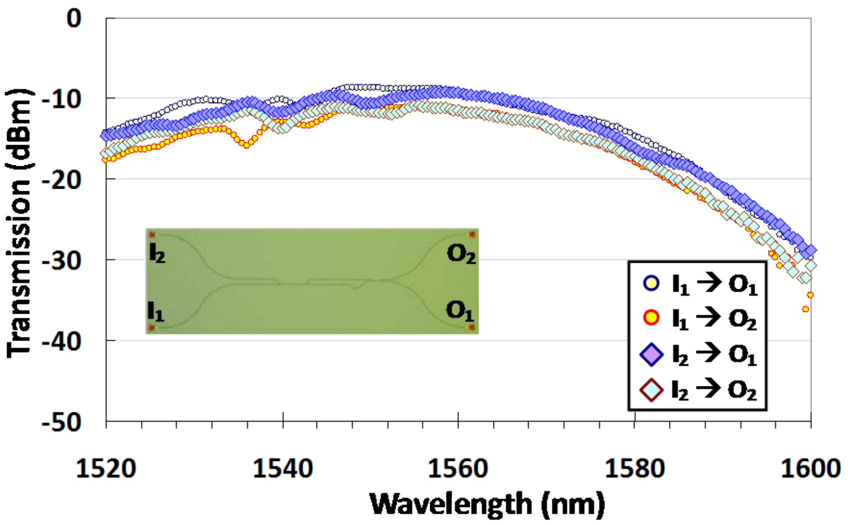


**Supplementary Figure 5**: **The microscope picture of sample 2 and the result of light test.** The splitting ratio of the beam-splitter was 50:50. The coupling loss was 10dB, and the modemux crosstalk was -25dB.

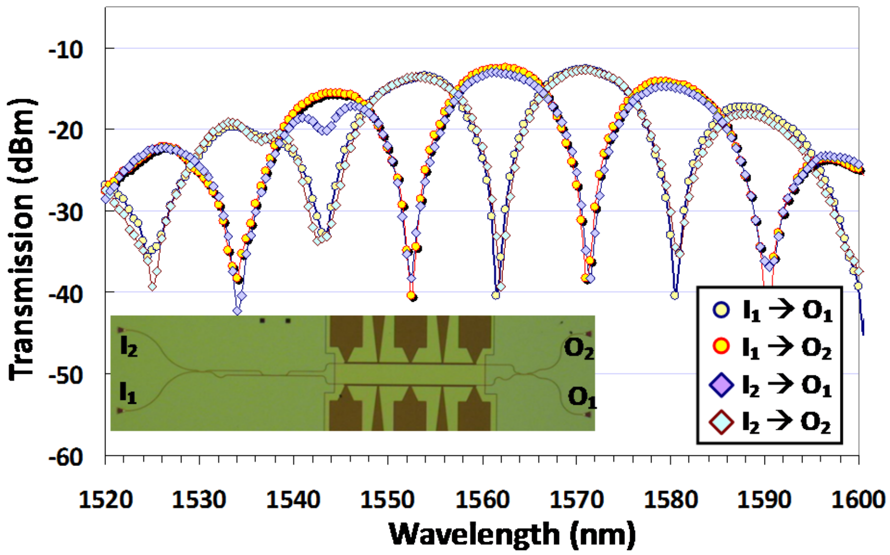


**Supplementary Figure 6**: **The microscope picture of sample 3 and the result of light test.** It was used for two-photon Machael-Zender interference. The phase between the two arms was adjusted by using a thermal-tuning method.

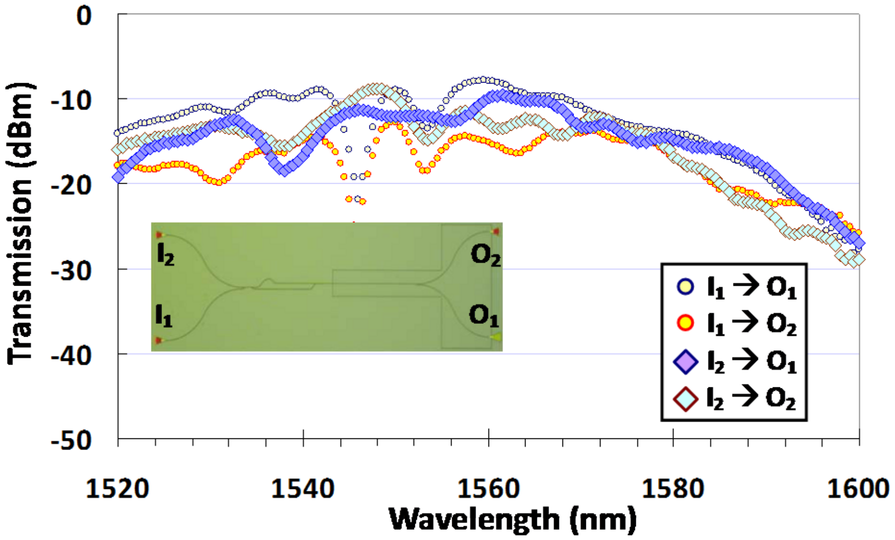


**Supplementary Figure 7**: **The microscope picture and the result of laser light test for sample 4.** The coupling loss was10dB and crosstalk was -25dB at 1560nm when light was coupled out at TE-type grating, while coupling loss was 12.5dB and crosstalk was -13dB at 1560nm when light was coupled out at TM-type grating. The modemux crosstalk was -25dB. The splitting ratio of the beam splitter was 50:50.
